# RadImageNet and ImageNet as Datasets for Transfer Learning in the Assessment of Dental Radiographs: A Comparative Study

**DOI:** 10.1007/s10278-024-01204-9

**Published:** 2024-07-24

**Authors:** Shota Okazaki, Yuichi Mine, Yuki Yoshimi, Yuko Iwamoto, Shota Ito, Tzu-Yu Peng, Taku Nishimura, Tomoya Suehiro, Yuma Koizumi, Ryota Nomura, Kotaro Tanimoto, Naoya Kakimoto, Takeshi Murayama

**Affiliations:** 1 Department of Medical Systems Engineering, Graduate School of Biomedical and Health Sciences, Hiroshima University, Hiroshima, Japan; 2 Project Research Center for Integrating Digital Dentistry, Hiroshima University, Hiroshima, Japan; 3 Department of Orthodontics and Craniofacial Developmental Biology, Graduate School of Biomedical and Health Sciences, Hiroshima University, Hiroshima, Japan; 4 Department of Pediatric Dentistry, Graduate School of Biomedical and Health Sciences, Hiroshima University, Hiroshima, Japan; 5 School of Dentistry, College of Oral Medicine, Taipei Medical University, Taipei, Taiwan; 6 Department of Genomic Oncology and Oral Medicine, Graduate School of Biomedical and Health Science, Hiroshima University, Hiroshima, Japan; 7 Department of Oral and Maxillofacial Radiology, Graduate School of Biomedical and Health Sciences, Hiroshima University, Hiroshima, Japan

**Keywords:** Deep Learning, Machine Learning, Panoramic Radiography, Cephalometry

## Abstract

Transfer learning (TL) is an alternative approach to the full training of deep learning (DL) models from scratch and can transfer knowledge gained from large-scale data to solve different problems. ImageNet, which is a publicly available large-scale dataset, is a commonly used dataset for TL-based image analysis; many studies have applied pre-trained models from ImageNet to clinical prediction tasks and have reported promising results. However, some have questioned the effectiveness of using ImageNet, which consists solely of natural images, for medical image analysis. The aim of this study was to evaluate whether pre-trained models using RadImageNet, which is a large-scale medical image dataset, could achieve superior performance in classification tasks in dental imaging modalities compared with ImageNet pre-trained models. To evaluate the classification performance of RadImageNet and ImageNet pre-trained models for TL, two dental imaging datasets were used. The tasks were (1) classifying the presence or absence of supernumerary teeth from a dataset of panoramic radiographs and (2) classifying sex from a dataset of lateral cephalometric radiographs. Performance was evaluated by comparing the area under the curve (AUC). On the panoramic radiograph dataset, the RadImageNet models gave average AUCs of 0.68 ± 0.15 (*p* < 0.01), and the ImageNet models had values of 0.74 ± 0.19. In contrast, on the lateral cephalometric dataset, the RadImageNet models demonstrated average AUCs of 0.76 ± 0.09, and the ImageNet models achieved values of 0.75 ± 0.17. The difference in performance between RadImageNet and ImageNet models in TL depends on the dental image dataset used.

**Supplementary Information:**

The online version contains supplementary material available at 10.1007/s10278-024-01204-9.

## Introduction

Artificial intelligence (AI) solutions could transform dental care and daily clinical practice. In particular, deep learning (DL), a branch of AI, is hoped to have the potential to assist practitioners with fast and accurate diagnosis [1, 2]. Convolutional neural networks (CNNs), a type of DL, have exhibited superior performance in image analysis to date in multiple dental imaging modalities [3, 4]. However, the performance of CNN relies heavily on large-scale labeled datasets for training, which poses significant challenges in medical image analysis [5]. The acquisition of medical image data is challenging and expensive, requiring advanced technology and expertise for labeling [5]. Moreover, data privacy and data sharing concerns further complicate this process [6]. These factors impede the widespread application and optimal performance of CNNs in medical image analysis, making it difficult to construct robust and generalizable models [7].

To overcome these barriers, transfer learning (TL) has been increasingly recognized as an effective strategy in the medical field [5]. TL is an alternative approach to full training of DL models from scratch, which utilizes a neural network pre-trained on a very large dataset and then fine-tuned for specific tasks using smaller, specialized datasets [8–10]. The origin of TL is from cognitive research and uses the idea that knowledge can be transferred between related tasks to improve performance on new tasks [11]. In other words, TL can transfer knowledge gained from large-scale data to solve different problems.

TL has been recognized as particularly suitable for medical image and signal analysis, as it achieves high performance with a small amount of labeled data for fine-tuning pre-trained networks [12, 13]. An additional advantage is the acceleration of the training process compared to learning from scratch. Moreover, it can mitigate the risk of overfitting that often occurs when working with small datasets. Consequently, TL has received considerable attention as a crucial technique that expands the potential of DL in limited data environments and accelerates the practical implementation of AI in the medical field.

ImageNet, which is a publicly available large-scale dataset, is commonly used for TL-based image analysis [14]. Many studies have applied pre-trained models from ImageNet to clinical prediction tasks and have reported promising results [15]. However, some have questioned the effectiveness of using ImageNet, which consists solely of natural images (i.e., does not include medical images), for medical image analysis [16–18].

To bridge the domain gap between natural and medical images in TL, the East River Medical Imaging Group launched RadImageNet, which is a large-scale, diverse medical image dataset [19, 20]. RadImageNet comprises 1.35 million annotated major medical images, including computed tomography (CT), magnetic resonance imaging (MRI), and ultrasound (US) imaging. Researchers at the Biomedical Engineering and Imaging Institute at the Icahn School of Medicine at Mount Sinai have demonstrated that TL using RadImageNet outperforms TL using ImageNet for both classification and segmentation tasks in medical images [19].

TL-based approaches using ImageNet have been extensively reported in dental research to diagnose radiographic images [18, 21]. However, it is unclear whether the use of natural image datasets is the best choice for dental imaging modalities. Hence, we aim to evaluate whether models pre-trained on RadImageNet could achieve superior performance on classification tasks in dental imaging modalities compared with ImageNet pre-trained models.

## Methods

### Study Design

This study design was approved by the Ethical Committee for Epidemiology of Hiroshima University (Approval Number: E2022-0211). All dental radiographs were acquired at Hiroshima University Hospital (Hiroshima, Japan). The ethical committee waived the requirement for informed consent by gaining consent using the opt-out method.

In this study, we conducted a binary classification task to evaluate the performance of TL under different conditions. We prepared two dental imaging datasets for TL (Supplementary Fig. 1). One dataset included panoramic radiographs of 200 children in the early mixed dentition stage, aged between 6 years 0 months and 9 years 6 months, acquired between April 2011 and March 2020. The panoramic radiograph dataset consisted of 100 images with no dental anomalies and 100 images with single supernumerary teeth. All panoramic radiographs were recorded using Hyper-X or SOLIO XZ systems (Asahi Roentgen Ind. Co., Ltd, Kyoto, Japan) [21]. The other dataset included lateral cephalometric radiographs of 400 patients aged between 5 years 8 months and 24 years 11 months (200 female, 200 male), acquired between April 2009 and March 2022. All lateral cephalometric radiographs were recorded in DICOM format using a cephalometric scanner (CX-150 W; Asahi Roentgen Ind. Co., Ltd., Kyoto, Japan). The original image resolution was 1648 × 1980 pixels, and the images were resized to 224 × 224 pixels [22].

**Fig. 1:**
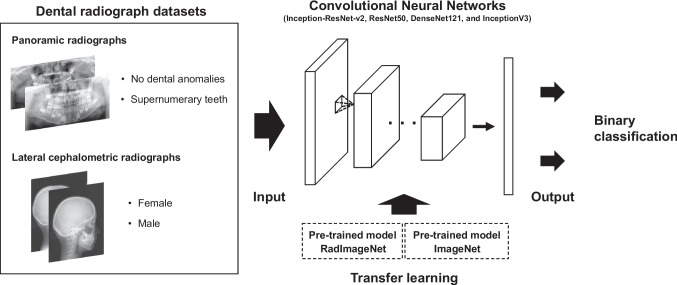
Schematic illustration of the comparison of classification performance between RadImageNet pre-trained models and ImageNet pre-trained models on two dental imaging datasets

As pre-trained models, we used four CNNs trained from scratch based on RadImageNet and ImageNet, reported by Mei et al. [19]. The RadImageNet pre-trained models and source code are publicly available at https://github.com/BMEII-AI/RadImageNet*.* The models employed were networks with the same architecture as Inception-ResNet-v2 [23], ResNet50 [24], DenseNet121 [25], and InceptionV3 [26]. We evaluated the classification performance of RadImageNet and ImageNet pre-trained models for TL using two dental imaging datasets. The tasks were (1) classifying the presence or absence of supernumerary teeth from the panoramic radiograph dataset and (2) classifying sex from the lateral cephalometric radiograph dataset (Fig. 1). The datasets were randomly split into 75% training set, 10% validation set, and 15% test set.

### Transfer Learning with RadImageNet and ImageNet Pre-trained Models

All procedures were performed on a computer with Intel Core i7-11700K 3.60GHz CPU (Intel, Santa Clara, CA, USA), 32GB RAM and NVIDIA GeForce RTX 3090 24GB GPU (NVIDIA, Santa Clara, CA, USA). The models were implemented using Python and the Keras framework, with TensorFlow as the backend.

**Fig. 2:**
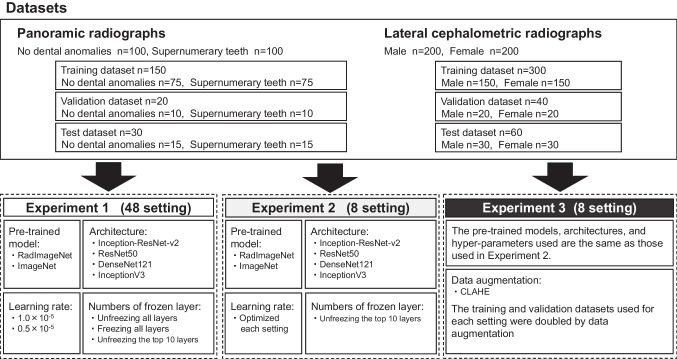
Flowchart of the experimental workflow

A flowchart of the study is showed in Fig. 2. Three experiments were conducted. In Experiment 1, minor modifications were made to the method proposed by Mei et al. [19] to simulate a total of 48 settings, thereby facilitating the evaluation of models for each dataset under various conditions. Four pre-trained models were trained with different learning rates and numbers of frozen layers. Unfreezing all layers, freezing all layers, and unfreezing the top 10 layers were performed at learning rates of 1.0 × 10^−5^ and 5.0 × 10^−6^. In Experiment 2, we explored conditions for optimizing models by unfreezing the top 10 layers of both RadImageNet and ImageNet, and fine-tuning the hyperparameters within each model. Based on the results of Mei et al.’s experiment and Experiment 1, the number of epochs and learning rate were fine-tuned, and the batch size and optimizer were set to the same conditions for all models (Table 1). In Experiment 3, the training and validation datasets were augmented by adapting the contrast-limited adaptive histogram equalization (CLAHE) technique [27, 28]. The hyperparameters are the same conditions as in Experiment 2. All experiments were performed using fivefold cross-validation.

**Table 1:**
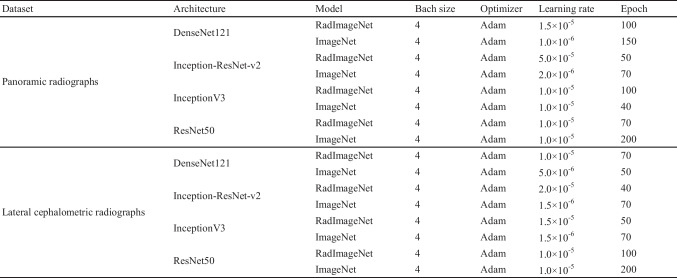
The experimental conditions on each dataset, architecture, and model

### Performance Metrics and Statistical Analysis

In Experiment 1, the methodology of Mei et al. [19] was adapted to compare the mean area under the receiver operating characteristic curve (AUC) values, along with their standard deviations, across 48 different settings between RadImageNet and ImageNet pre-trained models. The 95% confidence intervals (CIs) of AUC were evaluated, and the two-sided *p* values for the comparison of RadImageNet and ImageNet models were calculated. Statistical significance was defined as a *p* value less than 0.05.

The performance of the highest AUC scenario in ImageNet and RadImageNet with each dataset was assessed in terms of accuracy, precision, sensitivity, and F1 score. The performance metric values were evaluated using the following formulas:

$$Accuracy=\frac{TP+TN}{TP+FP+FN+TN}$$

$$Precision=\frac{TP}{TP+FP}$$

$$Sensitivity=\frac{TP}{TP+FN}$$

$$F1\; score=\frac{2\left(Sensitivity\times Precision\right)}{Sensitivity+Precision}$$

where TP, TN, FP, and FN are the true-positive, true-negative, false-positive, and false-negative counts.

In Experiment 2 and 3, the AUC, accuracy, precision, sensitivity, and F1 scores of the four optimized architectures were compared between RadImageNet and ImageNet pre-trained models.

## Results

**Fig. 3:**
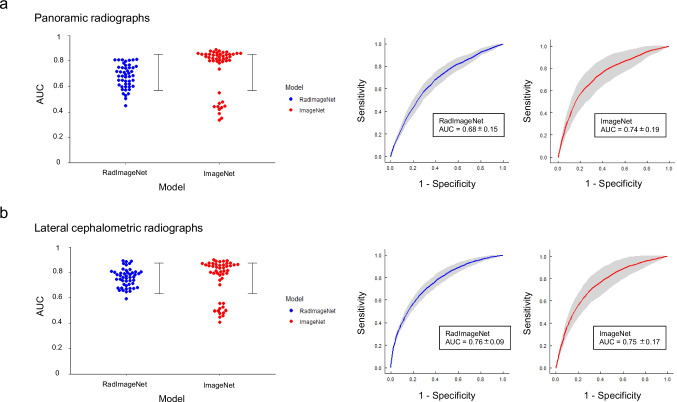
Two pre-trained models from RadImageNet and ImageNet were compared on two dental imaging datasets. **a** Panoramic radiograph dataset. **b** lateral cephalometric radiograph dataset. The beeswarm plots consist of 48 simulated experiments for the RadImageNet and ImageNet models. The error bars indicate the SD of all AUC values. Receiver operating characteristic curves demonstrate the average of 48 simulations of the RadImageNet and ImageNet models, respectively. The shaded areas indicate the SDs of Sensitivity and 1 – Specificity values

In Experiment 1, on the panoramic radiograph dataset, the RadImageNet models gave average AUCs of 0.68 ± 0.15 (SD) (*p* < 0.01), and the ImageNet models had values of 0.74 ± 0.19 (Fig. 3a). Conversely, on the lateral cephalometric dataset, the RadImageNet models demonstrated average AUCs of 0.76 ± 0.09, and the ImageNet models yielded values of 0.75 ± 0.17 (Fig. 3b). The difference in AUCs between RadImageNet and ImageNet and the associated 95% CIs were − 6.5% (− 9.5%, − 3.5%) and 0.7% (− 1.7%, 3.2%), on the panoramic radiograph dataset and the lateral cephalometric radiograph dataset, respectively.

As shown in Table 2, in the panoramic radiograph dataset, the highest AUC scenario of the RadImageNet models had accuracy, precision, sensitivity, and F1 scores of 74.7%, 78.7%, 74.7%, and 71.8%, respectively, while the highest AUC scenario of the ImageNet model had equivalent metrics of 71.3%, 67.3%, 93.3%, and 77.0%. In the lateral cephalometric radiograph dataset, the highest AUC scenario of the RadImageNet model gave accuracy, precision, sensitivity, and F1 scores of 80.3%, 88.2%, 72.0%, and 77.8%, respectively, while the highest AUC scenario of the ImageNet model had equivalent metrics of 80.3%, 77.6%, 86.7%, and 81.6%.

**Table 2:**
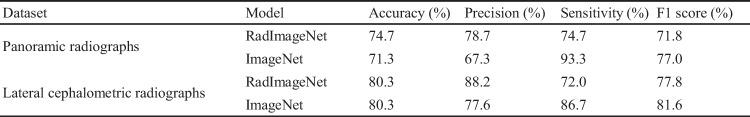
Performance metrics of the highest AUC scenario on each model and dataset

**Fig. 4:**
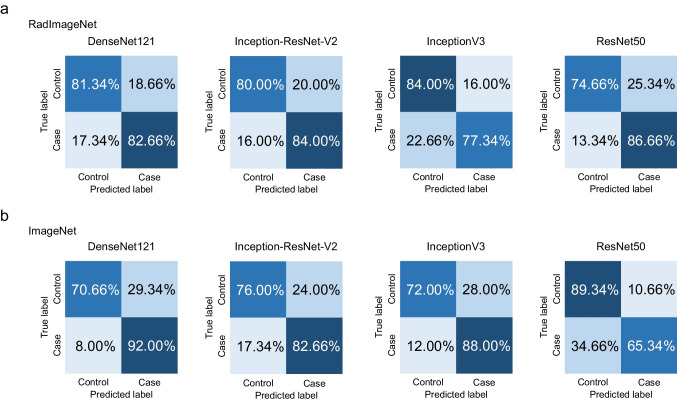
Confusion matrix for RadImageNet and ImageNet pre-trained models tested on the panoramic radiograph dataset in Experiment 2. Control, no dental anomalies; Case, supernumerary teeth

**Fig. 5:**
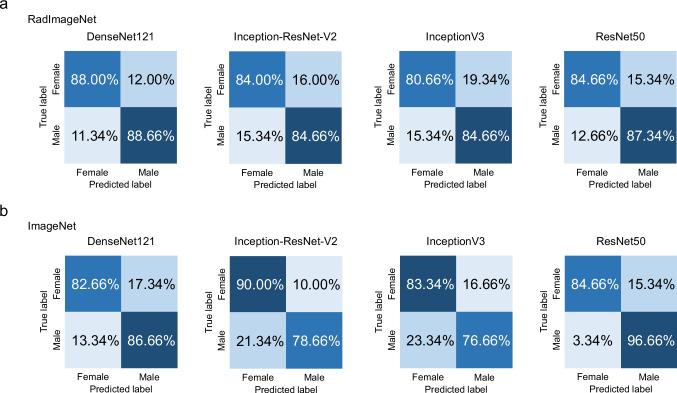
Confusion matrix for RadImageNet and ImageNet pre-trained models tested on the lateral cephalometric radiograph dataset in Experiment 2

The results of Experiment 2 are shown in Table 3 and Figs. 4 and 5. In the classification task for the panoramic radiograph dataset, the AUC values achieved by the RadImageNet model with optimized hyperparameters were 0.92 for DenseNet121, 0.86 for Inception-ResNet-v2, 0.86 for InceptionV3, and 0.89 for ResNet50, respectively. On the other hand, the AUC values achieved by the ImageNet model with optimized hyperparameters were 0.94 for DenseNet121, 0.89 for Inception-ResNet-v2, 0.86 for InceptionV3, and 0.89 for ResNet50, respectively. In the classification task for the lateral cephalometric radiograph dataset, the AUC values achieved by the RadImageNet model with optimized hyperparameters were 0.95 for DenseNet121, 0.92 for Inception-ResNet-v2, 0.91 for InceptionV3, and 0.93 for ResNet50, respectively. On the other hand, the AUC values achieved by the ImageNet model with optimized hyperparameters were 0.92 for DenseNet121, 0.90 for Inception-ResNet-v2, 0.85 for InceptionV3, and 0.96 for ResNet50, respectively.

**Table 3:**
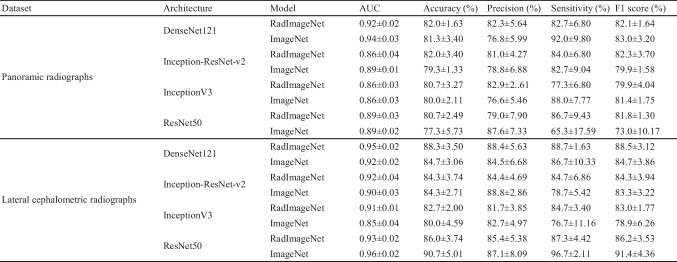
Performance metrics (mean ± standard deviation) for each dataset, architecture, and model in Experiment 2

**Fig. 6:**
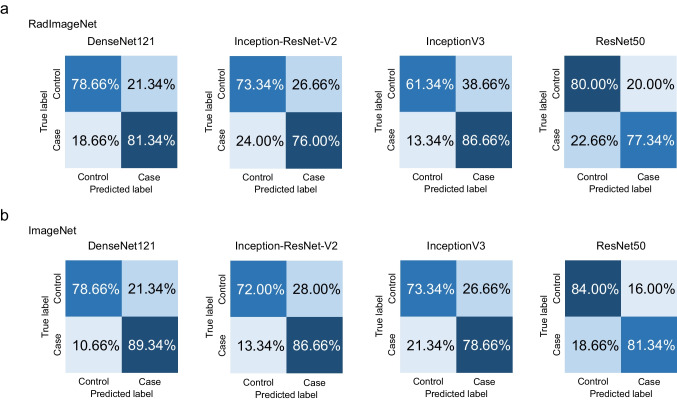
Confusion matrix for RadImageNet and ImageNet pre-trained models tested on the panoramic radiograph dataset in Experiment 3. Control, no dental anomalies; Case, supernumerary teeth

**Fig. 7:**
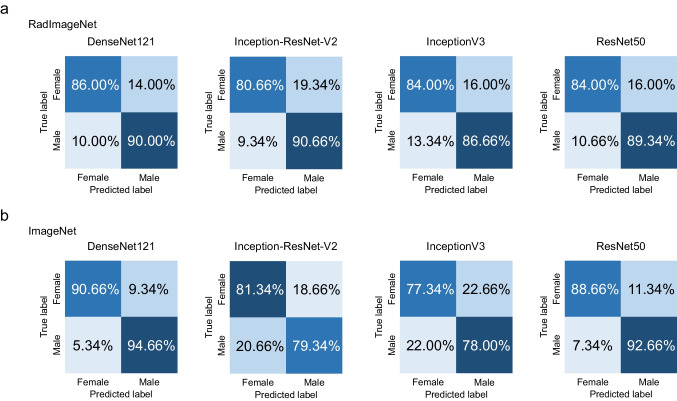
Confusion matrix for RadImageNet and ImageNet pre-trained models tested on the lateral cephalometric radiograph dataset in Experiment 3

The results of Experiment 3 are shown in Table 4 and Figs. 6 and 7. In the classification task for the panoramic radiograph dataset, the AUC values achieved by the RadImageNet model with optimized hyperparameters under the same condition as Experiment 2 ranged from 0.81 to 0.90. On the other hand, the AUC values obtained by the ImageNet model with optimized hyperparameters under the same condition as Experiment 2 ranged from 0.85 to 0.92. Experiment 3 showed no statistically significant difference in AUC values compared to Experiment 2. In the classification task for the lateral cephalometric radiograph dataset, the AUC values of the RadImageNet model with optimized hyperparameters under the same condition as Experiment 2 ranged from 0.93 to 0.95. On the other hand, the AUC values achieved by the ImageNet model with optimized hyperparameters under the same condition as Experiment 2 ranged from 0.85 to 0.96. In contrast to the panoramic radiograph dataset, data augmentation for the lateral cephalometric radiograph dataset yielded different results across architectures. Specifically, the ImageNet model for DenseNet121 showed higher AUC values when data augmentation was applied.

**Table 4:**
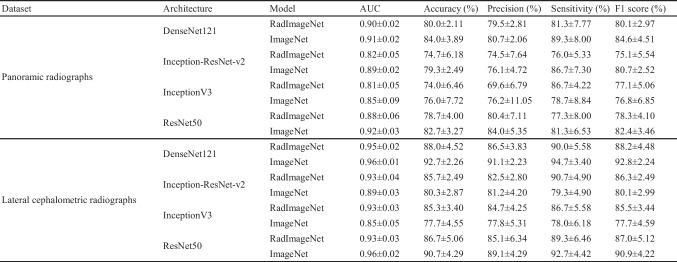
Performance metrics (mean ± standard deviation) for each dataset, architecture, and model in Experiment 3

## Discussion

Our study aimed to resolve issues specific to dental imaging in TL. Dental radiographs often have distinct characteristics, such as overlapping structures and variations in patient positioning, which can impact the effectiveness of pre-trained models. By directly comparing RadImageNet and ImageNet in this context, we provide insights into the applicability of these pre-training strategies for dental image analysis. RadImageNet, which motivated us to conduct this study, was reported in 2022 by Mei et al. [19] as a large dataset consisting of CT, MRI, and US images. Their study demonstrated the superiority of RadImageNet pre-trained models in classification tasks for multiple medical images across eight datasets ranging from 349 to 573,614 images. To assess the applicability of RadImageNet pre-trained models for dental images, we reproduced Mei et al.’s methodology and conducted additional experiments comparing RadImageNet and ImageNet pre-trained models on two dental image datasets.

Our research consisted of three main experiments. First, we followed Mei et al.’s method, evaluating models across 48 scenarios with varying architectures, freezing layers, and learning rates. Next, we compared the performance of RadImageNet and ImageNet pre-trained models after optimizing hyperparameters for each. Finally, we evaluated the pre-trained models by adapting CLAHE-based data augmentation in addition to the conditions of the second experiment. The number of datasets used in the third experiment roughly corresponds to the minimum number of datasets reported by Mei et al. [19].

Contrary to Mei et al.’s findings, our first and second experiments showed that the RadImageNet model was unable to achieve superior performance for both the panoramic radiograph dataset and the lateral cephalometric radiograph dataset with sagittal plane. Furthermore, our third experiment, which applied CLAHE-based data augmentation, failed to improve the classification performance of either the RadImageNet or ImageNet pre-training models. These results are more consistent with the findings of Suganuma et al. [29], who found that ImageNet pre-trained models outperformed RadImageNet pre-trained models in positron emission tomography/CT image segmentation tasks. Similarly, Nehary et al. [30] observed that for frame classification in lung ultrasound videos, models pre-trained on ImageNet outperformed those using RadImageNet, particularly for ResNet50 and DenseNet121 architectures. The variability in outcomes across different studies emphasize the complexity of TL in medical imaging. Dovile et al. [31], for instance, reported comparable performance between RadImageNet and ImageNet models but noted an inability to fully reproduce Mei et al.’s results regarding AUC. These discrepancies may be attributed to variations in experimental settings, including differences in image count and combinations of model architecture and hyperparameters. Remzan et al. [32] studied on brain tumor classification, and they found that RadImageNet pre-trained models did not consistently outperform ImageNet pre-trained models across all architectures and metrics. For example, their RadImageNet pre-trained DenseNet-121 model achieved an accuracy of 97.4% and an AUC of 99.83%, which was comparable to or slightly lower than results from existing studies using ImageNet pre-trained models, such as Kumar et al. [33], who achieved 97.08% accuracy using a ResNet-50 model. In contrast, Kihira et al. [34] demonstrated the superiority of RadImageNet pre-trained models in glioma segmentation tasks. Their RadImageNet pre-trained DenseNet121 model achieved a Dice similarity coefficient of 0.96 on the internal validation dataset and 0.93 on the external test dataset, outperforming ImageNet pre-trained models. The authors attribute this success to the higher similarity between RadImageNet and medical imaging tasks, as well as better reproducibility across datasets. Adding to this discourse, Zhang et al. [35] provided the evidence for the superiority of RadImageNet in classifying osteoporotic vertebral fractures from radiographic images. Their RadImageNet-based ResNet-50 model consistently outperformed the ImageNet-based model across multiple validation datasets, with statistically significant differences in macro-average AUC values. The authors attribute this success to RadImageNet’s specialization in medical imaging, which includes diverse anatomical structures that are more relevant to the task than the natural images in ImageNet. These findings collectively suggest that the effectiveness of RadImageNet versus ImageNet pre-training may depend on various factors, including the specific medical imaging modality, the nature of the classification task, the method of data presentation, and the characteristics of the target dataset. Our study, along with others in the field, contributes to the ongoing discussion about optimal pre-training strategies for medical image classification tasks.

Our study has several limitations that should be considered when interpreting the results. The relatively small size of our dental image datasets may limit the generalizability of our findings. Although we attempted to mitigate this through data augmentation techniques, larger datasets would provide more robust results. In addition, our study focused specifically on panoramic and lateral cephalometric radiographs, and the results may not be generalizable to other dental imaging modalities or general medical imaging tasks. Our experiments were also limited to classification tasks, and the relative performance of RadImageNet and ImageNet pre-training may differ for other tasks, such as segmentation or detection in dental imaging. Although we attempted to optimize the hyperparameters, the large hyperparameter space means that we may not have found the optimal configuration for each model. This is an indication of the difficulty of TL in medical imaging and the need for careful consideration of model selection and tuning. These limitations emphasize the need for further research with larger and more diverse dental imaging datasets, as well as exploration of additional model architectures and tasks. Future studies should aim to address these limitations in order to provide more comprehensive insights into the application of TL in dental imaging.

## Conclusion

In conclusion, our research highlights the need for careful empirical evaluation when selecting pre-training strategies for dental imaging tasks. The performance differences observed between pre-trained RadImageNet and ImageNet models in different dental imaging tasks suggest that the optimal pre-training strategy may be context dependent. This emphasizes the importance of conducting comprehensive evaluations when selecting pre-training approaches for specific dental imaging applications. By considering factors such as the nature of the target dataset, the specific requirements of the dental imaging task, and the method of data presentation and analysis, researchers and practitioners can make more informed decisions when developing AI-based dental diagnostic tools.

Future work should focus on several key areas to advance the application of TL in dental imaging. These include collecting larger and more diverse dental datasets, exploring multi-task learning scenarios, investigating advanced domain adaptation techniques, developing explainable AI models for dental imaging, ablation study, and conducting longitudinal studies. By addressing these areas, future research can contribute to the development of more effective, reliable, and clinically applicable TL solutions for dental imaging.

This approach will help optimize the performance of TL models in various dental imaging applications, thus improving patient care and assisting dental professionals in their diagnostic and treatment planning processes. To more efficiently apply TL in the dental field, we will conduct a more detailed evaluation of RadImageNet by increasing the number and variety of target dental datasets, paving the way for more robust and generalizable AI solutions in dentistry.

## Supplementary Information

Below is the link to the electronic supplementary material.

Supplementary file1 (PPTX 12685 KB)

## Data Availability

The data that support the findings of this study are available from the corresponding author upon reasonable request.
