# Supplementary material for: RadImageNet and ImageNet as Datasets for Transfer Learning in the Assessment of Dental Radiographs: A Comparative Study
Source: J Imaging Inform Med. 2024 Jul 24;38(1):534–44. doi: 10.1007/s10278-024-01204-9 (PMC11811346; doi:10.1007/s10278-024-01204-9)
Supplement: Supplementary file 1 — Supplementary file1 (PPTX 12685 KB) [file 10278_2024_1204_MOESM1_ESM.pptx]

## Slide 1
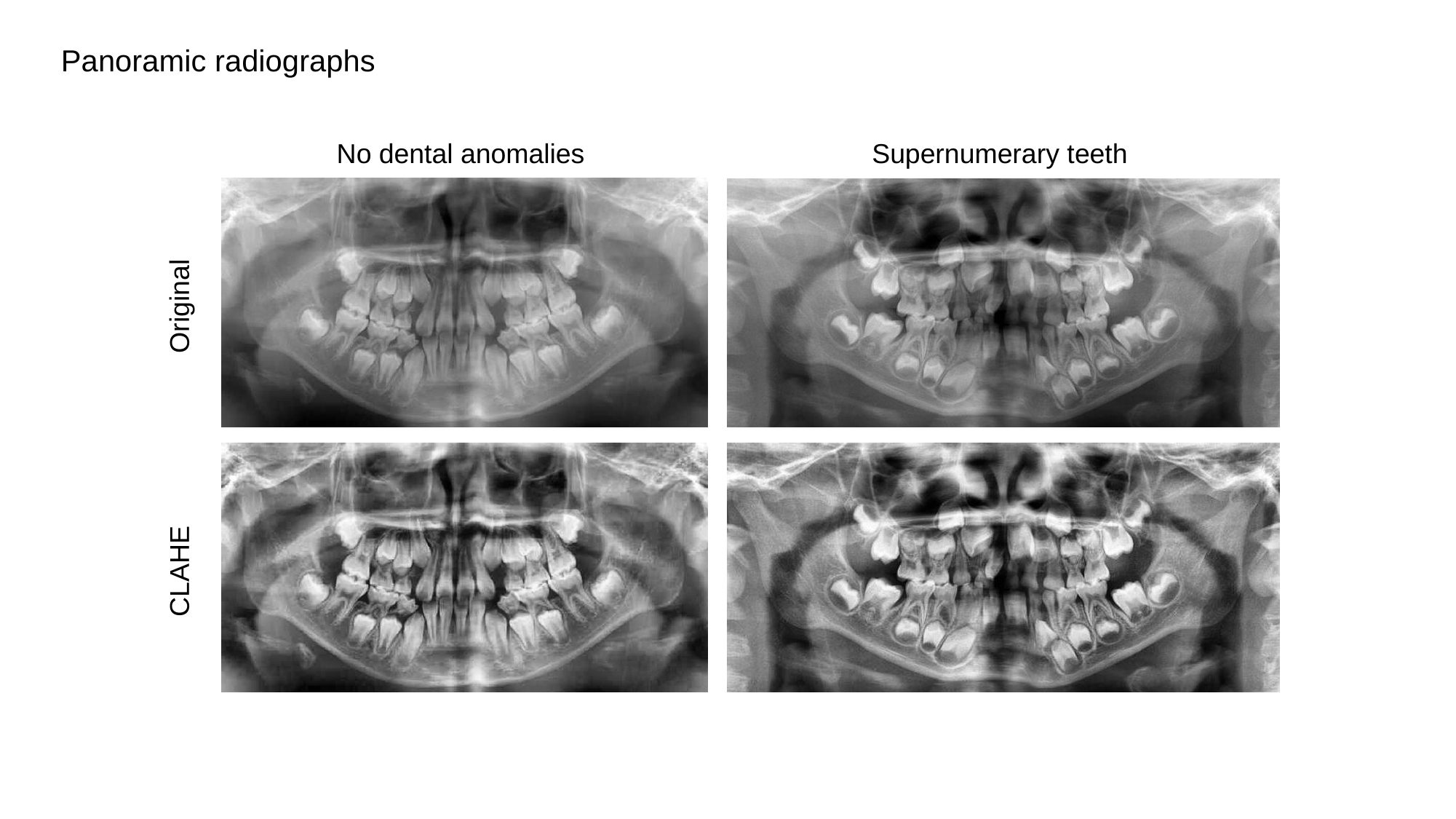

Panoramic radiographs
No dental anomalies
Supernumerary teeth
Original
CLAHE

## Slide 2
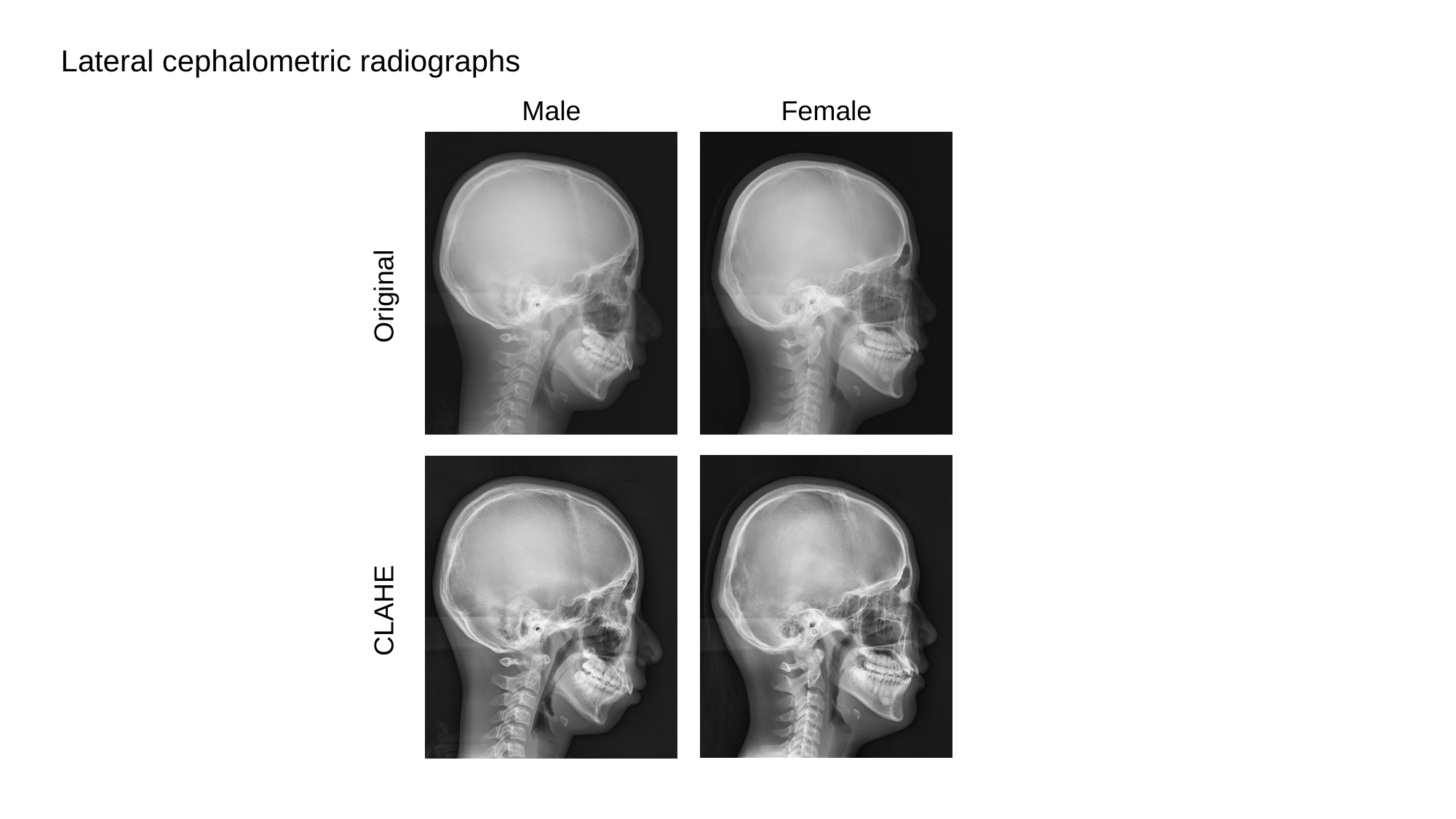

Lateral cephalometric radiographs
Male
Female
Original
CLAHE
